# S100A10 protein expression is associated with oxaliplatin sensitivity in human colorectal cancer cells

**DOI:** 10.1186/1477-5956-9-76

**Published:** 2011-12-30

**Authors:** Sayo Suzuki, Yasuko Yamayoshi, Akito Nishimuta, Yusuke Tanigawara

**Affiliations:** 1 Department of Clinical Pharmacokinetics and Pharmacodynamics, School of Medicine, Keio University, 35 Shinanomachi, Shinjuku-ku, Tokyo 160-8582, Japan; 2 Hospital Pharmacy, Keio University Hospital, 35 Shinanomachi, Shinjuku-ku, Tokyo 160-8582, Japan

**Keywords:** oxaliplatin, biomarker, S100A10, colorectal cancer, SELDI-TOF MS

## Abstract

**Background:**

Individual responses to oxaliplatin (L-OHP)-based chemotherapy remain unpredictable. The objective of our study was to find candidate protein markers for tumor sensitivity to L-OHP from intracellular proteins of human colorectal cancer (CRC) cell lines. We performed expression difference mapping (EDM) analysis of whole cell lysates from 11 human CRC cell lines with different sensitivities to L-OHP by using surface-enhanced laser desorption/ionization time-of-flight mass spectrometry (SELDI-TOF MS), and identified a candidate protein by liquid chromatography/mass spectrometry ion trap time-of-flight (LCMS-IT-TOF).

**Results:**

Of the qualified mass peaks obtained by EDM analysis, 41 proteins were differentially expressed in 11 human colorectal cancer cell lines. Among these proteins, the peak intensity of 11.1 kDa protein was strongly correlated with the L-OHP sensitivity (50% inhibitory concentrations) (*P *< 0.001, *R^2 ^*= 0.80). We identified this protein as Protein S100-A10 (S100A10) by MS/MS ion search using LCMS-IT-TOF. We verified its differential expression and the correlation between S100A10 protein expression levels in drug-untreated CRC cells and their L-OHP sensitivities by Western blot analyses. In addition, S100A10 protein expression levels were not correlated with sensitivity to 5-fluorouracil, suggesting that S100A10 is more specific to L-OHP than to 5-fluorouracil in CRC cells. S100A10 was detected in cell culture supernatant, suggesting secretion out of cells.

**Conclusions:**

By proteomic approaches including SELDI technology, we have demonstrated that intracellular S100A10 protein expression levels in drug-untreated CRC cells differ according to cell lines and are significantly correlated with sensitivity of CRC cells to L-OHP exposure. Our findings provide a new clue to searching predictive markers of the response to L-OHP, suggesting that S100A10 is expected to be one of the candidate protein markers.

## Background

Oxaliplatin (L-OHP) is a third-generation platinum compound, used as a key drug for the treatment of colorectal cancer (CRC). L-OHP and bolus/infusional 5-fluorouracil (5-FU) combined with folinic acid (FOLFOX) have yielded high response rates (≈50%) and good overall survival [1-4]. However, approximately half of all patients who receive FOLFOX gain no benefit, despite the usual risk of toxicity. The ability to predict a patient's response to L-OHP-based regimens would thus facilitate the rational use of chemotherapy for CRC.

Several predictive markers of the response to platinum-based chemotherapy have been proposed on the basis of various mechanisms of chemoresistance to platinum drugs, including DNA-repair pathways and detoxification pathways, as well as drug metabolism and transport [5]. Genomic polymorphisms participating in nucleotide excision repair pathways, such as excision repair cross-complementing rodent repair deficiency, complementation group 1 (*ERCC1*) and xeroderma pigmentosum group D (*XPD*, also known as *ERCC2*), and the glutathione-S-transferase family of isozymes in detoxification pathways are considered potential predictors of clinical outcomes in patients given L-OHP-based chemotherapy [6-9]. However, how to predict the clinical response of CRC to L-OHP-based chemotherapy remains unclear [10].

Protein expression profiles reflect the intracellular biological status more directly than gene markers because gene expression provides no information on post-translational modifications. Recently, the ProteinChip^® ^System, using surface-enhanced laser desorption/ionization time-of-flight mass spectrometry (SELDI-TOF MS), has been widely used to obtain protein profiles of biological samples [11]. This system is high-throughput, requires only small samples, and can comprehensively analyze hundreds of proteins directly from crude samples [12]. Moreover, SELDI-TOF MS is well suited for analyzing low-molecular weight proteins (< 20 kDa), which are abundant in physiologically important proteins, such as cytokines, chemokines, or fragments of larger proteins.

We aimed to identify protein biomarker candidates predictive of L-OHP sensitivity. By proteomic approaches including SELDI technology, we have identified a candidate protein using CRC cell lines.

## Results

### L-OHP sensitivity

**Figure 1:**
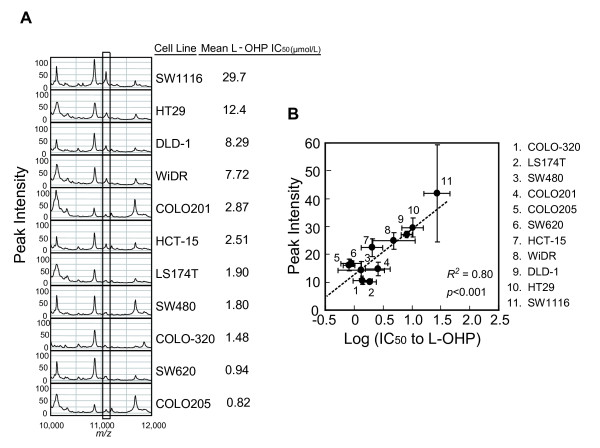
**L-OHP sensitivity and candidate peak selection**. (A) Protein expression profiles of each cell line on CM10 array at pH 4.5. The candidate peak is enclosed by the rectangle. (B) Peak intensity of the 11.1 kDa protein in 11 CRC cell lines strongly correlates with L-OHP sensitivity. The peak intensity and IC_50 _value of each cell line are plotted as means ± S.D. (peak intensity, n = 3; IC_50_, n = 3 or 4).

The 50% inhibitory concentration (IC_50_) values of 11 CRC cell lines with different chemosensitivities to L-OHP were measured. The evaluated IC_50 _values (μM) (mean ± S.D.) were as follows: COLO205, 0.822 ± 0.236; SW620, 0.937 ± 0.332; COLO-320, 1.48 ± 0.51; SW480, 1.80 ± 1.62; LS174T, 1.90 ± 0.44; HCT15, 2.51 ± 0.61; COLO201, 2.87 ± 1.67; WiDR, 7.72 ± 4.67; DLD-1, 8.29 ± 1.85; HT29, 12.4 ± 5.7; SW1116, 29.7 ± 13.6 (Figure 1A).

### Candidate biomarker selection

We obtained the protein profiles of 11 human CRC cell lines other than HCT116 which was reserved for subsequent validation. Of the qualified mass peaks obtained by expression difference mapping (EDM) analysis, 41 proteins were differentially expressed in 11 human colorectal cancer cell lines (Figure 1A). Of these, the peak intensity of the 11.1 kDa protein strongly correlated with the sensitivity to L-OHP (*P *< 0.001, *R^2 ^*= 0.80; Figure 1B). This correlation was independent of cell type and cell growth rate, because the doubling times of the 10 cell lines were similar (20.8-28.5 h). The doubling time of SW1116 was 105 hr (data not shown). These data suggest that this protein is a candidate biomarker that strongly correlates with sensitivity to L-OHP.

### Protein characterization

**Figure 2:**
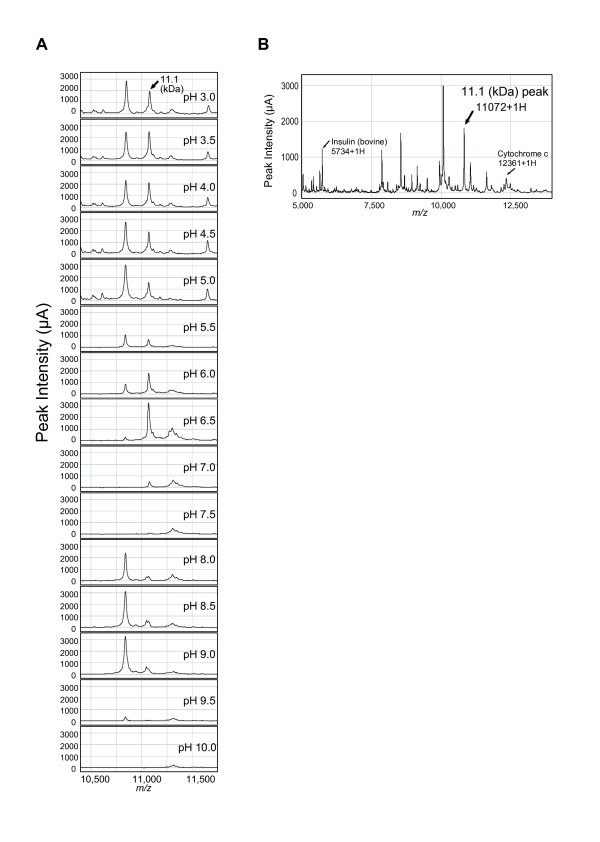
**Characterization of the 11.1 kDa protein**. (A) Estimation of pI of the 11.1 kDa protein. The affinity of the 11.1 kDa protein (indicated by the arrow) to a CM10 array changed with a pH range of 3.0-10.0. (B) Estimation of experimental molecular mass of the 11.1 kDa protein (indicated by the arrow). Bovine insulin (5733.5 Da) and equine cytochrome C (12361.0 Da) were calibrants for internal calibration.

We first characterized the protein by SELDI retentate chromatography mass spectrometer (SELDI-RCMS) using cell lysates of HT29. This protein had a high affinity to CM10 array in acidic environment and was undetectable on the array at pH 7.0-7.5, indicating a pI in this pH range (Figure 2A). The experimental molecular mass was estimated as *m/z *(mass-to-charge ratio) 11,072 by internal calibration (Figure 2B).

### Protein identification

**Figure 3:**
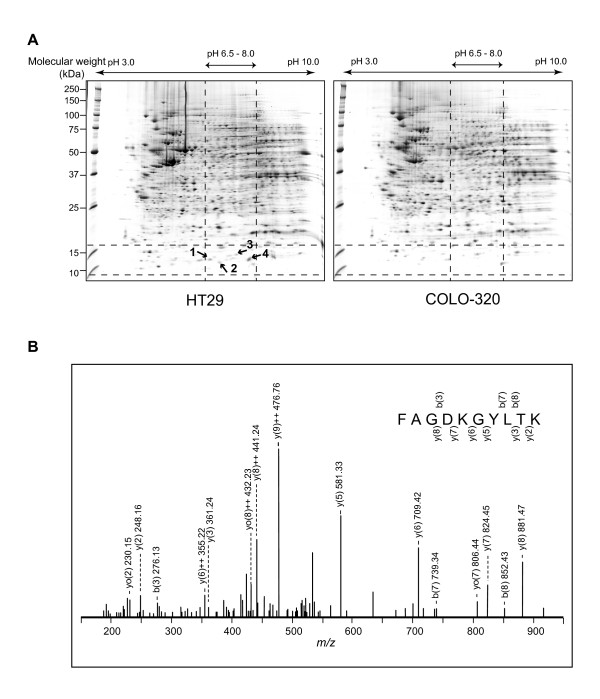
**Identification of the 11.1 kDa protein**. (A) 2-DE maps of whole cell lysates from HT29 (left panel) and COLO-320 (right panel), which showed high and low expression of the 11.1 kDa protein, respectively. The 4 spots indicated by the arrows were selected on the basis of the experimental molecular mass (*m/z *11,072) and experimental pI (7.0-7.5) of the candidate protein. (B) The MS/MS spectrum of tryptic-digested *m/z *550.29 recorded from spot 4. The amino acid sequence of this peptide fragment is highlighted on the spectrum.

Figure 3A shows the Coomassie brilliant blue (CBB)-stained 2D gels of cell lysates from HT29 and COLO-320. Based on the experimental pI and mass determined, we obtained 4 spots from the HT29-gel, which showed over 3-fold differences in densitometric volumes in HT29-gel as compared with those in COLO-320-gel. Three proteins obtained by in-gel trypsin digestion of the spots were identified (Table 1). The 11.1 kDa protein was identified as Protein S100-A10 (UniProtKB/Swiss-Prot: P60903) (S100A10), which was derived from spot 4 (Figure 3B). The theoretical molecular weight (11,072 Da) and theoretical pI (7.31) of S100A10 (UniProtKB/Swiss-Prot, http://www.expasy.org) were consistent with the experimental values determined by SELDI-RCMS.

**Table 1:** List of identified proteins.

Mass spectrum data: protein data of all 2-DE spots selected, peptide data from spot 4 and sequence coverage of spot 4 (Protein S100-A10).								
**Protein Data**								

		**Physical data**			**MS/MS ion search results**		**Characterization of 11.1 kDa protein by SELDI-TOF MS**	
					
		**Swiss-Prot**			**In gel digestion from 2-DE**			
				
**Spot (s)**	**Protein name**	**Swiss-Prot Accession No**.	**Theoretical molecular weight using Expasy tool (Da)**	**Theoretical pI using Expasy tool**	**Sequence coverage** **(%)**	**Mowse score**	**Observed molecular mass** **(*m/z*)**	**Observed pI**
		
1	Protein S100-A11	P31949	11741	6.55	26	78		
2	No hit	-	-	-	-	-		
3	Beta-2 microglobulin	P61769	11731	6.08	17	65		
4	Protein S100-A10	P60903	11072	7.31	10	38	11072	7.0-7.5

**Peptide data from spot 4 (Protein S100-A10)**								
	Start - End		Observed	Mr (expt)	Mr (calc)	Delta	Miss	Sequence
	
	18 - 27		550.29	1098.57	1098.57	0.00	1	K.FAGDKGYLTK.E

**Sequence coverage**								
	Matched peptide is underlined 1 PSQMEHAMET MMFTFHK**FAG DKGYLTK**EDL RVLMEKEFPG FLENQKDPL 51 VDKIMKDLDQ CRDGKVGFQS FFSLIAGLTI ACNDYFVVHM KQKGKK							

Mowse score > 30 indicates identity or extensive homology (p < 0.05). Search parameters: MS/MS ion search, enzyme: trypsin, fixed modificaton: carbamidomethyl (C), variable modification: oxidation (M), peptide tolerance: ± 0.05 Da, fragment mass tolerance: ± 0.05 Da, max missed cleavage: 1.

### Validation of the identified protein by Western blot analysis

**Figure 4:**
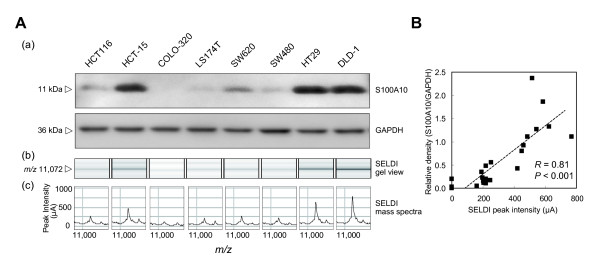
**Comparison of Western blot densitometry and SELDI peak intensity**. (A) A Western blot illustrating the differential expression of S100A10 derived from cell lysates of 8 CRC cell lines (a). SELDI-TOF MS gel view (b) and mass peak (c) illustrating the differential expression of the protein at *m/z *11,072 in cell lysates from 8 CRC cell lines. The results are representative of three separate experiments. (B) The results of Western blot densitometry of S100A10 significantly correlated with the SELDI peak intensity at *m/z *11,072. Twenty-four experimental data points (3 separate experiments with each of 8 cell lines) are plotted.

To confirm the identified protein and to validate the results of SELDI-TOF MS analysis, Western blot analyses of Protein S100A10 in whole cell lysates from 8 CRC cell lines (HCT15, COLO-320, LS174T, SW620, SW480, HT29, DLD-1 and HCT116) were performed. HCT116, which was not used in the candidate search study, was newly introduced in this validation study. Western blot densitometry of S100A10 was consistent with the peak intensity detected on SELDI-TOF MS (Figure 4A). Figure 4B shows a high correlation between the peak intensity at *m/z *11,072 detected on SELDI-TOF MS and the Western blot densitometry for S100A10 (*P *< 0.001, *R *= 0.81). We identified the candidate protein as S100A10 and confirmed that S100A10 was differentially expressed by the CRC cell lines.

### Correlation between S100A10 protein expression levels and sensitivity to L-OHP or 5-fluorouracil (5-FU)

**Figure 5:**
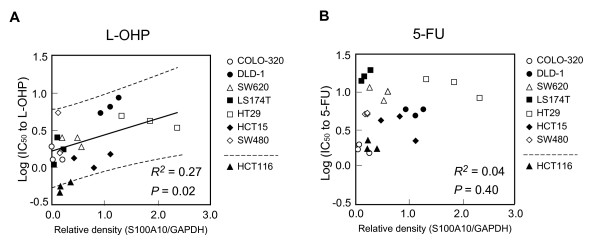
**Relationship between S100A10 protein expression levels and sensitivity to L-OHP or 5-FU**. The S100A10 protein expression level is associated with L-OHP sensitivity (*P *= 0.02, *R^2 ^*= 0.27) (A), but not with 5-FU sensitivity (*P *= 0.40, *R^2 ^*= 0.04) (B). The relationship between the sensitivity to L-OHP or 5-FU and S100A10 protein expression levels in 7 cell lines which were used in the candidate search were examined by the linear regression analysis. Twenty-one experimental data points (3 separate experiments with each of 7 cell lines) of the index data set and 3 experimental data points of HCT116 as a testing sample are plotted. Linear regression line (solid line) and 95% prediction interval (dotted line) are depicted.

To confirm the results of candidate search study by SELDI-TOF MS analysis, we investigated the relationship between the sensitivity to L-OHP and S100A10 protein expression levels quantified by Western blot densitometry in 7 cell lines, which were used in the candidate search study, as the index data set. The cells with higher S100A10 protein expression levels tended to exhibit lower chemosensitivity to L-OHP (Figure 5A). The data points of newly introduced HCT116 were plotted in lower limit of the 95% prediction interval, showing its high chemosensitivity to L-OHP with a low protein expression of S100A10, consisting with our findings. On the other hand, there was no significant correlation between S100A10 protein expression levels and IC_50 _values for 5-FU (*P *= 0.40, *R^2 ^*= 0.04, Figure 5B), demonstrating that the S100A10 protein expression level does not reflect chemosensitivity to 5-FU, the antitumor mechanism of which differs from that of platinum-containing compounds. The IC_50 _values (μM) (mean ± S.D.) of 8 cell lines for 5-FU were as follows: HCT116, 1.84 ± 0.29; HCT15, 3.59 ± 1.29; COLO-320, 1.81 ± 0.34; LS174T, 16.5 ± 2.8; SW620, 9.72 ± 2.00; SW480, 4.95 ± 0.16; HT29, 12.0 ± 3.5; and DLD-1, 4.73 ± 0.96.

### Presence of S100A10 in the culture supernatant

**Figure 6:**
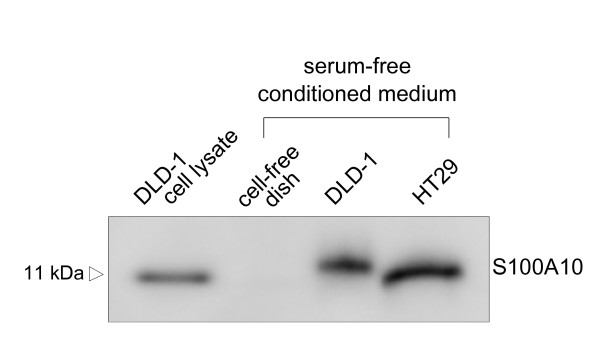
**Detection of S100A10 in serum-free conditioned medium (SFCM)**. Western blotting of SFCM from HT29 or DLD-1. DLD-1 whole cell lysate (10 μg) was used as a positive control, and conditioned medium from a 'cell-free' dish treated with the same protocol was used as a negative control.

To assess the extracellular secretion of S100A10, Western blotting was performed for serum-free conditioned medium (SFCM) incubated with HT29 or DLD-1, which exhibit high protein expression levels of intracellular S100A10. The result demonstrated the presence of S100A10 in culture supernatant (Figure 6). Cell viability was > 80% after incubation with serum-free medium (data not shown).

## Discussion

Predictive markers of chemotherapeutic response are urgently needed to improve the outcomes of cancer treatment. Predictive markers of the response to L-OHP have not yet been established [5], and clinically available protein markers of drug-response are also limited [13]. In this study, by proteomic approach, we found that intracellular S100A10 protein expression levels were significantly correlated with sensitivity of CRC cells to L-OHP, providing a new clue to predictive markers of the response to L-OHP.

The SELDI peak intensity of S100A10 varied more than 4-fold among various CRC cell lines (Figure 1), and Western blot analysis confirmed the differential expression of S100A10 (Figure 4). This is the first time to report the differential protein expression of S100A10 in a variety of CRC cell lines. These data also confirmed the quantitative accuracy of SELDI peak intensity, indicating the usefulness of ProteinChip technology for further clinical validation of S100A10 in the next step of our research with high sample throughput.

S100A10 is a member of the S100 family of proteins. It has been shown to interact with a variety of proteins, including plasma membrane-resident receptors and channels such as serotonin 1B (5-HT_1B_) receptor [14-17], indicating that S100A10 is an active regulator and/or is involved in the trafficking of cellular/membrane proteins which lead to various biological functions. S100A10 mRNA, S100A10 protein, or both have been found in many types of cells, tissues, and tumors [18-23]. S100A10 has been identified as a plasminogen receptor, suggesting its promotion of angiogenesis and tumor metastasis [24,25]. S100A10 has thus attracted considerable attention for its role in cancer development. However, our present results demonstrate for the first time that S100A10 correlates with the chemosensitivity of CRC cells to L-OHP.

In this study we indicate that intracellular S100A10 in CRC cells is associated with cell survival after L-OHP exposure, not 5-FU exposure (Figure 1, Figure 5), suggesting that S100A10 is a potential biomarker more specific to L-OHP. *ERCC1 *has been suggested to play an important role in resistance to platinum-based chemotherapy [5]. However, the UK MRC FOCUS (Fluorouracil, Oxaliplatin, CPT-11: Use and Sequencing) clinical trial, the largest randomized biomarker trial in metastatic CRC to date, reported no significant association of response with *ERCC1, ERCC2*, glutathione-S-trasnferase-P1 (*GSTP1*), or other candidate biomarkers that had previously shown promise [10]. The value of *ERCC1 *as a predictive marker of the response to L-OHP-based chemotherapy remains uncertain [26].

Mechanisms of S100A10 involvement in chemoresistance are unknown at this moment. However, a few studies have reported an association of S100A10 with cell viability. S100A10 interacts with BAD (Bcl-xL/Bcl-2 associated death promoter), a death enhancer, and blunts its pro-apoptotic activity [27]. S100A10 is induced by nerve growth factor, and increased S100A10 levels promote the proliferation of PC12 cells, a pheochromocytoma cell line [28]. These previous results agree with our finding that CRC cells with high intracellular S100A10 expression enhanced cell survival after L-OHP exposure. S100A10 interacts with cytosolic phospholipase A2 (cPLA2, 85-kDa) and inhibits its activity, resulting in decreased release of arachidonic acid (AA) [29]. S100A10 also interacts with 5-HT_1B _receptor and modulates its function [17]. Both AA and serotonin play important role in CRC physiology [30-33]. S100A10 may be involved in releasing pro-inflammatory cytokines. Down-regulation of S100A10 in human macrophages inhibits the plasmin-induced release of pro-inflammatory cytokines such as interleukin-6 [34], which has been suggested to promote cell growth and apoptosis-escape of colon cancer [35,36].

A number of factors were reported to regulate S100A10 expression [37], leading to another speculation that intracellular S100A10 is a surrogate of other active molecules related to cell survival after exposure to anticancer agents, as follows. Down-regulation of caveolin-1, which has recently attracted attention for its potential role in chemoresistance [38,39], reduces intracellular S100A10 protein expression and its localization to caveolae in HCT116, although the mechanisms involved are unknown [40].

Most S100A10 is tightly associated with the dimers of annexin A2 (ANXA2), forming an (ANXA2)_2_-(S100A10)_2 _heterotetramer [41-43]. ANXA2 is a member of the annexin family which has been reported to have multiple functions [44-47], and requires S100A10 for its action and translocation to the cell surface [48]. These previous observations indicate the possibility that intracellular S100A10 protein reflects the handling of other active molecules related to cell survival after exposure to anticancer agents.

S100A10 appears to merit further investigation as a potential predictive biomarker of the response of CRC to L-OHP. We have also demonstrated the presence of S100A10 in cell culture supernatant (Figure 6), suggesting that S100A10 undergoes extracellular secretion. In addition, recently, S100A10 was detected in human serum and the list of plasma protein [49,50], thereby allowing blood level monitoring of S100A10 for further clinical validation as a biomarker for the L-OHP sensitivity.

Thus, molecular backgrounds of S100A10 described in previous reports are partly consistent with our hypothesis that S100A10 expression level may reflect the chemosensitivity, from a view of chemosensitivity. However, mechanisms of S100A10 involvement in chemoresistance are unknown and the reason why S100A10 is more predictive of L-OHP sensitivity than 5-FU sensitivity is not clarified in this study. The present study was designed to identify and characterize S100A10 associated with the chemosensitivity. The molecular mechanisms of S100A10 as a predictive biomarker of L-OHP response will be addressed in subsequent studies.

## Conclusions

We have demonstrated by proteomic approaches including SELDI technology that intracellular S100A10 protein expression levels in drug-untreated CRC cells differ according to cell lines and are significantly correlated with sensitivity of CRC cells to L-OHP exposure. Our results provide new primary findings for searching predictive markers of the response to L-OHP, suggesting that S100A10 is expected to be one of the candidate protein markers. To confirm this hypothesis, further clinical validation and functional analysis to elucidate the underlying biological mechanisms are necessary.

## Methods

### Agents and antibodies

L-OHP was kindly provided by Yakult Honsha, Co., Ltd. (Tokyo, Japan). 5-FU was purchased from Sigma-Aldrich (St. Louis, MO, USA). Purified mouse anti-human annexin II Light Chain (S100A10) mAb was obtained from BD Biosciences (Mississauga, ON, Canada), and anti-human GAPDH mAb was obtained from Applied Biosystems (Foster City, CA, USA). All other chemicals and reagents were of the highest purity available.

### Cell cultures

Twelve human CRC cell lines were used. DLD-1, HT29, SW480, SW1116, WiDR, and HCT116 were purchased from the European Collection of Cell Cultures (Salisbury, UK), and SW620 was purchased from the American Type Culture Collection (Manassas, VA, USA). COLO205, HCT-15, and LS174T were provided by the Cell Resource Center for Biomedical Research, Tohoku University (Sendai, Japan). COLO201 was provided by the Japanese Collection of Research Bioresource (Tokyo, Japan), and COLO-320 was provided by RIKEN Bio-Resource (Tsukuba, Japan). The cells were cultured in RPMI 1640 medium supplemented with 10% FBS and 2 mM glutamine at 37°C in humidified air containing 5% CO_2_. Exponentially growing cells were used.

### Chemosensitivity tests and IC_50 _determination

The cells were plated at the following densities in 96-well plates: COLO205, HCT15, HT29, and WiDR, 1000 cells/well; COLO201, DLD-1, LS174T, HCT116, and SW620, 1500 cells/well; COLO-320 and SW480, 3000 cells/well; and SW1116, 5000 cells per well. The cells were cultured for 24 h before addition of various concentrations of L-OHP, ranging from 0 to 1000 μM. Cell viability after incubation with L-OHP for 48 h was assayed using the CellTiter96^® ^AQueous One Solution Cell Proliferation Assay (MTS assay, Promega Corporation, Madison, WI, USA) according to the manufacturer's protocol. The sensitivity of cells to L-OHP was evaluated by determining the IC_50 _values by fitting the concentration-survival curve to a logistic function:

$$\text{P}=1∕\left(1+\text{exp}\left(-\alpha -\beta \cdot \text{log}\text{D}\right)\right)$$

P: ratio to drug-free control

D: drug concentration

IC_50 _= 10^(-α/β)^

IC_50 _values of cells treated with 5-FU were also evaluated with the MTS assay after 72 h of exposure to 5-FU in concentrations ranging from 0 to 10 mM. The IC_50 _values for L-OHP or 5-FU were log transformed for normal distribution, and the log_10_IC_50 _values were used for further statistical analysis.

### Sample preparation

Cells were washed 3 times with cold PBS and lysed in a lysis buffer containing 9 M urea, 2% CHAPS, 1 mM dithiothreitol, and protease inhibitor cocktail (Sigma-Aldrich, St. Louis, MO, USA). After incubation on ice for 10 min followed by sonication on ice, the lysates were centrifuged at 15,000 × g for 30 min at 4°C, and the supernatant was collected. The protein concentration was determined by DC Protein Assay (Bio-Rad Laboratories, Hercules, CA, USA), and aliquots were quickly frozen in liquid nitrogen and stored at -80°C until analysis.

### SELDI ProteinChip array preparation

Weak cation-exchange ProteinChip CM10 arrays (Bio-Rad Laboratories, Hercules, CA, USA) were used for protein profiling. Protein concentrations of the cell lysates were adjusted to 5 mg/mL by adding lysis buffer and then diluted to 1 mg/mL with binding/washing buffer (50 mM sodium acetate [pH 4.5]). CM10 arrays were equilibrated with 150 μL binding/washing buffer for 5 min, and incubated with 100 μL of diluted sample. After 1 h, each spot was washed 3 times with 150 μL binding/washing buffer and rinsed twice with 400 μL of distilled water. After air-drying, 0.5 μL of a saturated solution of sinapic acid in 50% (v/v) acetonitrile containing 0.1% (v/v) trifluoroacetic acid was applied twice to the surface of each spot and dried.

### SELDI-TOF MS analysis

The prepared CM10 arrays were analyzed using a ProteinChip SELDI Reader, Model PBS IIc (Ciphergen Biosystems, Fremont, CA, USA), for EDM analysis in the candidate selection study, and Model PCS-4000 personal edition (Bio-Rad Laboratories, Hercules, CA, USA) for the subsequent confirmation study. Mass spectrometry profiles were generated using 108 laser shots with a laser intensity of 220 and a detector sensitivity of 8 for PBS IIc, or 265 laser shots with a laser intensity of 3000 nJ for PCS-4000. The *m/z *of each protein was determined with the use of externally calibrated standards (ProteoMass™ Peptide & Protein MALDI-MS Calibration Kit, Sigma-Aldrich, St. Louis, MO, USA). Peaks were auto-detected at an *m/z *of 2,000 to 30,000 and a signal-to-noise ratio of > 5 for PBS IIc or a valley depth of 5 for PCS-4000. Spectra were baseline-subtracted and normalized to the total ion current. All calculations were performed using ProteinChip data manager software (Bio-Rad Laboratories, Hercules, CA, USA).

### Correlation analysis between protein expression and chemosensitivity

To identify protein biomarkers, we screened proteins whose peak intensity was associated with the sensitivity to L-OHP, by investigating the relations between IC_50 _values for L-OHP and each peak intensity on EDM analysis across 11 cell lines (excluding HCT116) by linear regression analysis. Candidate peaks were then selected according to the following criteria: *P *value < 0.05 and coefficient of determination (*R*^2^; *R*, Pearson correlation coefficient) > 0.5.

### Identification of candidate biomarker proteins

#### Characterization of target proteins

A SELDI-RCMS on CM10 arrays was used to estimate the experimental pI of the target protein [51]. Briefly, whole cell lysates from HT29 were diluted with binding buffers (pH 3.0-10.0 in 0.5 increments) and analyzed using the PCS-4000. To estimate the experimental molecular mass of the target protein, internal calibration was carried out using bovine insulin (5733.5 Da) and equine cytochrome C (12361.0 Da) of ProteoMass™ Peptide & Protein MALDI-MS Calibration Kit (Sigma-Aldrich, St. Louis, MO, USA), which bookended the target protein.

#### Two-dimensional electrophoresis (2-DE)

To identify the target protein, we used HT29 and COLO-320, which show high or low expression of this protein, respectively. After desalting and concentrating the cell lysates (250 μg) by acetone precipitation, cell extracts dissolved in isoelectric focusing (IEF) buffer containing 6 M urea, 2 M thiourea, 3% CHAPS, 1% Triton X-100 and DeStreak reagent (GE Healthcare, Little Chalfont, UK) were rehydrated in Immobiline DryStrip gel (pH 3-10 non-linear, GE Healthcare, Little Chalfont, UK) for 12 h. Then, IEF was performed at 150 V for 1 h, followed by 5,000 V ramping for 2.5 h and 5,000 V IEF for 15 h. Before 2-dimensional PAGE (2D-PAGE), the strip gel was equilibrated in sample buffer (6 M urea, 20% glycerol, 2% DTT, 2% SDS, 375 mM Tris-HCl, pH 8.8) for 45 min. 2D-PAGE was performed using polyacrylamide gradient gel (10-18%). SDS-PAGE gels were stained with CBB G-250 (Bio-Rad Laboratories, Hercules, CA, USA). Images were acquired with a GS-800 calibrated densitometer (Bio-Rad Laboratories, Hercules, CA, USA) and analyzed with PDQuest 7.2.0 software (Bio-Rad Laboratories, Hercules, CA, USA).

#### MS/MS ion search and protein identification

Protein identification of gel spots was performed by LCMS-IT-TOF (Shimadzu, Kyoto, Japan). The gel pieces of interests were excised from the gel, and in-gel digestion of protein was performed. Tryptic peptides were separated via reversed-phase liquid chromatography/mass spectrometry using DiNa nanoLC (KYA Tech Corporation, Tokyo, Japan) for analytical separation on a New Objective PicoFrit BetaBasic C18 column (100 mm × 75 μm). Mass spectrometric analysis ([+] ESI) was carried out on an LCMS-IT-TOF with argon gas for ion cooling and CID experiments. Tandem mass spectrometry data were obtained in a data-dependent manner. A Mascot search engine (Matrix Science, Boston, MA, USA) was used for protein database searching. Search parameters are described in Table 1. Proteins with statistically significant MASCOT/Mowse score (> 30), indicating identity or extensive homology (p < 0.05), were considered to be identified.

### Preparation of SFCM

HT29 or DLD-1 cells were plated at a density of 1 × 10^7 ^cells per 10-cm dish and incubated for 24 h. The medium was changed, and after additional incubation for 24 h, the cells were washed 6 times with serum-free fresh medium (SFM) and received 8 mL of SFM per 10-cm dish. After 24 h incubation with SFM, the resultant conditioned medium containing secreted proteins was centrifuged to remove cell debris and concentrated 1000-fold by ultrafiltration with an Amicon^® ^Ultra Centrifugal Filter and a 3,000 Dalton molecular mass cutoff spin column (Millipore Corporation, Billerica, MA, USA). As a negative control, a 'cell-free' dish was treated with the same protocol.

### Western blot analysis

Total cell lysates (5 μg protein) and concentrated SFCM (15 μL) were fractionated by SDS-PAGE. The separated proteins were transferred electrophoretically to PVDF membranes by using an iBlot^® ^Dry Blotting System (Invitrogen, Carlsbad, CA, USA). After blocking, the blots were probed with a 1:5000 dilution of mouse anti-human S100A10 primary antibody or a 1:4000 dilution of mouse anti-human GAPDH primary antibody and developed with a WesternBreeze^® ^Chemiluminescent Western Blot Immunodetection Kit according to the manufacturer's instructions (Invitrogen, Carlsbad, CA, USA). GAPDH was used as a loading control. Protein bands were visualized with an LAS 4000 mini imaging system (FUJIFILM, Tokyo, Japan) and analyzed with Multi Gauge software Ver 3.0 (FUJIFILM, Tokyo, Japan).

### Statistical analysis

Statistical analyses were performed using SPSS software 17.0J for Windows (SPSS, Chicago, IL, USA). To evaluate relations between two variables, correlation analysis and regression analyses were used. *P *values < 0.05 were considered statistically significant. All tests were two-sided.

## List of abbreviations

L-OHP: oxaliplatin; CRC: colorectal cancer; EDM: expression difference mapping; SELDI-TOF MS: surface-enhanced laser desorption/ionization time-of-flight mass spectrometry; LCMS-IT-TOF: liquid chromatography/mass spectrometry ion trap time-of-flight; IC_50_: 50% inhibitory concentration; ERCC1: excision repair cross-complementing rodent repair deficiency; complementation group 1; XPD/ERCC2: xeroderma pigmentosum group D; GSTP1: glutathione-S-trasnferase-P1; SELDI-RCMS: SELDI retentate chromatography mass spectrometer; *m/z*: mass-to-charge ratio; CBB: Coomassie brilliant blue; 5-FU: 5-fluorouracil; SFCM: serum-free conditioned medium; ANXA2: annexin A2; mAb: monoclonal antibody; SFM: serum-free fresh medium.

## Competing interests

Yusuke Tanigawara received a research grant from Yakult Honsha Co., Ltd. Sayo Suzuki, Yasuko Yamayoshi and Akito Nishimuta declare no competing interests.

## Authors' contributions

YT was responsible for planning and designing the study and data interpretation. SS performed the experiments and the data analysis and wrote the manuscript. YY participated in SELDI-TOF MS analysis. AN participated in cell culture. All authors read and approved the final manuscript.
